# Genetic and behavioral adaptation of *Candida parapsilosis* to the microbiome of hospitalized infants revealed by in situ genomics, transcriptomics, and proteomics

**DOI:** 10.1186/s40168-021-01085-y

**Published:** 2021-06-21

**Authors:** Patrick T. West, Samantha L. Peters, Matthew R. Olm, Feiqiao B. Yu, Haley Gause, Yue Clare Lou, Brian A. Firek, Robyn Baker, Alexander D. Johnson, Michael J. Morowitz, Robert L. Hettich, Jillian F. Banfield

**Affiliations:** 1 Department of Plant and Microbial Biology, University of California, Berkeley, CA USA; 2 Graduate School of Genome Science and Technology, The University of Tennessee, Knoxville, TN USA; 3 Chemical Sciences Division, Oak Ridge National Laboratory, Oak Ridge, TN USA; 4 Department of Microbiology and Immunology, Stanford University School of Medicine, Stanford, CA 94305 USA; 5 Chan Zuckerberg Biohub, San Francisco, CA USA; 6 Department of Microbiology and Immunology, University of California, San Francisco, San Francisco, CA USA; 7 Department of Surgery, University of Pittsburgh School of Medicine, Pittsburgh, PA USA; 8 Division of Newborn Medicine, Magee-Womens Hospital of UPMC, Pittsburgh, PA USA; 9 Department of Earth and Planetary Science, University of California, Berkeley, CA USA; 10 Department of Environmental Science, Policy, and Management, University of California, Berkeley, CA USA; 11 Earth Sciences Division, Lawrence Berkeley National Laboratory, Berkeley, CA USA

**Keywords:** Microbial eukaryotes, Metagenomics, Genome-resolved metagenomics, Strain-tracking, Hospital microbiome, Neonatal intensive care unit, Premature infants, Candida

## Abstract

**Background:**

*Candida parapsilosis* is a common cause of invasive candidiasis, especially in newborn infants, and infections have been increasing over the past two decades. *C. parapsilosis* has been primarily studied in pure culture, leaving gaps in understanding of its function in a microbiome context.

**Results:**

Here, we compare five unique *C. parapsilosis* genomes assembled from premature infant fecal samples, three of which are newly reconstructed, and analyze their genome structure, population diversity, and in situ activity relative to reference strains in pure culture. All five genomes contain hotspots of single nucleotide variants, some of which are shared by strains from multiple hospitals. A subset of environmental and hospital-derived genomes share variants within these hotspots suggesting derivation of that region from a common ancestor. Four of the newly reconstructed *C. parapsilosis* genomes have 4 to 16 copies of the gene RTA3, which encodes a lipid translocase and is implicated in antifungal resistance, potentially indicating adaptation to hospital antifungal use. Time course metatranscriptomics and metaproteomics on fecal samples from a premature infant with a *C. parapsilosis* blood infection revealed highly variable in situ expression patterns that are distinct from those of similar strains in pure cultures. For example, biofilm formation genes were relatively less expressed in situ, whereas genes linked to oxygen utilization were more highly expressed, indicative of growth in a relatively aerobic environment. In gut microbiome samples, *C. parapsilosis* co-existed with *Enterococcus faecalis* that shifted in relative abundance over time, accompanied by changes in bacterial and fungal gene expression and proteome composition.

**Conclusions:**

The results reveal potentially medically relevant differences in Candida function in gut vs. laboratory environments, and constrain evolutionary processes that could contribute to hospital strain persistence and transfer into premature infant microbiomes.

**Video abstract**

**Supplementary Information:**

The online version contains supplementary material available at 10.1186/s40168-021-01085-y.

## Background

Candida species are the most common cause of invasive fungal disease [1, 2]. A variety of Candida species cause candidiasis and are recognized as a serious public health challenge, especially among immunocompromised and hospitalized patients [3, 4]. Historically, *Candida albicans* most commonly has been recognized as the cause of candidiasis, and as a result, is the focus of the majority of Candida research [4–6]. However, *Candida parapsilosis*, despite being considered less virulent than *C. albicans*, is the Candida species with the largest increase in incidence since 1990 [6]. Given important differences in the biology of *C. albicans* compared to non-albicans species, more research on non-albicans Candida species, especially the subset that poses a serious health risk, is needed [4].

*C. parapsilosis* is often a commensal member of the gastrointestinal tract and skin [6, 7]. Passage from hospital workers’ hands to immunocompromised patients is thought to be a common cause of opportunistic infection in hospital settings [8]. *C. parapsilosis* infections of premature infants are of particular concern. Indeed, *C. parapsilosis* is the most frequently isolated fungal organism in many neonatal intensive care units (NICUs) in the UK [3] and is responsible for up to one-third of neonatal Candida bloodstream infections in North America [9]. Adding to the concern is the limited number of antifungal drugs and the increasing prevalence of antifungal drug resistance in Candida species. An estimated 3–5% of *C. parapsilosis* are resistant to fluconazole, the most commonly applied antifungal [10]. The recent emergence of multidrug-resistant *Candida auris* with its resultant high mortality rate [11] serves as a warning regarding the potential for outbreaks of multidrug-resistant *C. parapsilosis*. Therefore, understanding behavior of *C. parapsilosis*, both as a commensal organism and opportunistic pathogen, is incredibly important.

A challenge that complicates understanding of the medically relevant behavior of Candida in the human microbiome is that the hosts used in model infection systems (e.g., rat or murine mucosa) are not natural hosts to Candida species. Study of Candida in these models relies on some form of predisposition of the animal by occlusion, immunosuppression, surgical alteration, or elimination of competing microbial flora [1]. Pure culture experiments, an alternative to model system studies, are often the most accessible way to study Candida. However, the lack of a microbial community context is a large caveat, considering bacteria could influence the nutrition, metabolism, development, and evolution of eukaryotes. Indeed, other microbial eukaryotes have been shown to be dramatically influenced by their surrounding microbial communities. Choanoflagellates, the closest known living relative of animals, live in aquatic environments and feed on bacteria by trapping them in their apical collar [12]. The Choanoflagellate S*alpingoeca rosetta* is primarily a unicellular organism but formation of multicellular rosettes is induced by a sulphonolipd (RIF1) and inhibited by a sulfonate-containing lipid, both produced by the bacterium *Algoriphagus machipongonensis* [13]*.* Furthermore, the bacterium *Vibrio fischeri* produces a chondroitinase, EroS, capable of inducing sexual reproduction in *S. rosetta* [14]. Together, these results demonstrate the influence that bacteria can exert on the morphology, development, and evolution of microbial eukaryotes.

There is more direct evidence motivating study of *C. parapsilosis* functioning in situ. For instance, *Caenorhabditis elegans* model of polymicrobial infection experiments showed that *C. albicans* exhibits complex interactions with *Enterococcus faecalis*, a bacterial human gut commensal and opportunistic pathogen. In this context, *C. albicans* and *E. faecalis* negatively impact one another’s virulence [15], suggesting a mechanism that promotes commensal behavior in a gut microbial community context. The decrease in *C. albicans* virulence was attributed to inhibition of hyphal morphogenesis and biofilm formation by proteases secreted by *E. faecalis* [15] as well as E. faecalis capsular polysaccharide [16]. No research has investigated *C. parapsilosis* in a microbial community context.

An alternative to studying Candida species in animal models or laboratory cultures is to use an untargeted shotgun sequencing approach (genome-resolved metagenomics). DNA is extracted from fecal or other samples and sequenced. The subsequent DNA sequences are assembled, and metagenome-assembled genomes (MAGs) are reconstructed. Much work of this type has focused on the bacterial members of the human microbiome; however, recently developed methods such as EukRep [17] enable reconstruction of eukaryotic genomes from metagenomes with greater consistency, including genomes of *Candida* species [18]. The availability of genomes enables evolutionary studies and the application of other ‘omics’ approaches, such as transcriptomics, proteomics, and metabolomics, making it possible to go beyond metabolic potential to study activity in situ. Although there are limitations related to establishing causality via experimentation, the approaches can provide insights into metabolism and changes in metabolism linked to shifts in community composition in human-relevant settings.

Here, we applied shotgun metagenomics, metatranscriptomics, and metaproteomics to investigate the behavior and evolution of Candida in the premature infant gut and hospital environment. Novel assembled *C. parapsilosis* and *C. albicans* genomes were reconstructed and the metagenomic data analyzed in terms of heterozygosity and population diversity. Due to the substantially less prior research on *C. parapsilosis* and the availability of *C. parapsilosis*-containing samples suitable for transcriptomics and proteomics, we focused our analyses on *C. parapsilosis* and identified genes and genomic regions under diversifying selection. Notably, we also identified instances of copy number gain of a gene involved in fluconazole resistance, pointing to a mechanism for hospital adaptation [19]. *C. parapsilosis* in situ transcriptomic and proteomic profiles were clearly distinct from profiles reported previously from culture settings. Substantial shifts in *C. parapsilosis* expression occurred with changes in microbiome composition over a few day period, suggesting the strong influence of bacterial community composition on *C. parapsilosis* behavior.

## Results

### Recovery of novel Candida strain genomes

A large dataset of a mixture of previously analyzed and newly generated infant gut and NICU shotgun metagenome samples were analyzed for the purpose of reconstructing novel Candida genomes (see the “Methods” section). Newly generated data includes fecal samples collected and sequenced from two infants targeted for having documented Candida infections during fecal collection. Previously analyzed data includes fecal samples collected from 163 premature infants primarily during the first 30 days of life (DOL) (full range of DOL 5–121), with an average of 7 samples per infant. In addition, samples of the Neonatal intensive care unit (NICU) were taken from six patient rooms within the hospital housing the infants (Magee-Womens Hospital of UPMC, Pittsburgh, PA, USA). Finally, publicly available New York City subway shotgun metagenomes [20] were included after identifying Candida reads in one of the samples.

Candida genomes were assembled from samples containing > 2 Mbp of predicted eukaryotic DNA using a EukRep-based pipeline [17]; see the “Methods” section for details. Eight new, unique Candida genomes were assembled for this study (Table 1), five *C. albicans* genomes, and three *C. parapsilosis* genomes. Three additional Candida genomes were assembled but have been analyzed previously [18] along with the bacterial component of the samples [21] (see the “Methods” section), totaling in 11 Candida genomes reconstructed from infant gut and hospital room metagenomes. Nine of the 11 genomes were reconstructed from premature infant fecal samples; 1 genome was derived from a NICU room sample S2_005, and 1 from New York City Subway Samples [20]. Genomes representing new strains were named after their sample of origin. For comparison to isolate genomes, we analyzed 4 previously published *C. parapsilosis* and 51 *C. albicans* isolate genomes.

**Table 1:** Overview of Candida strain genomes used in this study

Genome	Genus	Species	Length	# Scaffolds	N50	BUSCO comp.	Year sampled	Sample type	Reference
C1_006	Candida	Parapsilosis	11852211	191	108686	92	2017	Infant fecal metagenome	This study
N3_182	Candida	Parapsilosis	12563647	342	65710	94	2013	Infant fecal metagenome	Olm et al. 2019 [18, 21]
S2_005	Candida	Parapsilosis	11573959	1051	14507	93	2014	NICU metagenome	Olm et al. 2019 [18, 21]
NYC Subway	Candida	Parapsilosis	7420453	1285	6417	62	NA	NYC subway metagenome	This study
L2_023	Candida	Parapsilosis	4870205	2906	1700	35	2017	Infant fecal metagenome	This study
CDC317	Candida	Parapsilosis	13030174	9	2091826	93	NA	Clinical skin isolate	Butler et al. 2009 [22]
GA1	Candida	Parapsilosis	13025060	39	1114083	93	NA	Clinical human blood isolate	Pryszcz et al. 2013 [23]
CBS1984	Candida	Parapsilosis	13044404	25	962200	92	NA	Olive fruit isolate	Pryszcz et al. 2013 [23]
CBS6318	Candida	Parapsilosis	13050515	28	1691491	93	NA	Healthy skin isolate	Pryszcz et al. 2013 [23]
N1_023	Candida	Albicans	13456346	1675	15180	94	2012	Infant fecal metagenome	This study
N2_070	Candida	Albicans	13540857	1614	14761	93	2012	Infant fecal metagenome	This study
N5_264	Candida	Albicans	11647081	746	27434	85	2015	Infant fecal metagenome	This study
S3_003	Candida	Albicans	11972257	1049	14710	87	2017	Infant mouth metagenome	This study
S3_016	Candida	Albicans	10068784	802	19749	86	2018	Infant mouth, skin, and gut metagenome coassembly	This study
SP_CRL	Candida	Albicans	12561678	897	22840	91	NA	Infant fecal metagenome	Olm et al. 2019 [18, 21]

### Candida genomic variability

To characterize genomic variability in the strains of *C. albicans* and *C. parapsilosis* represented by metagenome-derived genomes, we identified single-nucleotide variants (SNVs) by mapping reads against completed reference genomes (strain SC5314 for *C. albicans* and CDC317 for *C. parapsilosis*). *C. albicans* genomes ranged from 3.2 to 9.9 heterozygous SNVs per kb (heterozygosity), whereas *C. parapsilosis* genomes ranged from 0.12 to 0.38 heterozygous SNVs per kb. Heterozygous SNVs were defined as SNVs with two or more alleles detected in a single sample, indicating different alleles between chromosomes. Thus, we infer that, compared to *C. albicans*, *C. parapsilosis* displays very low genetic variability between its diploid chromosome pair, which can be indicative of low genetic variability in the hospital environment and primarily asexual reproduction [24].

Low heterozygosity in *C. parapsilosis* genomes has been reported for previously sequenced genomes [23]. Interestingly, *C. parapsilosis* genomes derived from our fecal metagenomes showed even lower overall heterozygosity than pure culture reference genomes (Figure S1). In general, this would not be expected because within-sample population diversity due to sampling of a microbial community should inflate measures of genomic heterozygosity. Thus, the lower genomic heterozygosity may be reflective of infants being initially colonized by essentially a single *C. parapsilosis* genotype.

**Fig. 1:**
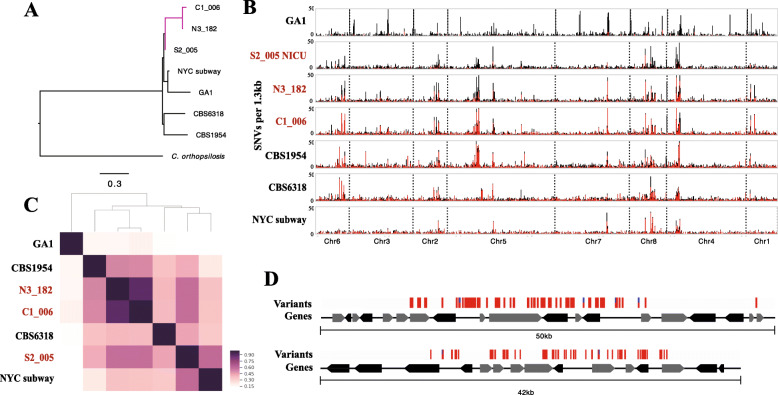
Analysis of *C. parapsilosis* genomic variability reveals a potential hospital associated population and the presence of SNV hotspots. **A** A phylogenetic tree of *C. parapsilosis* strains constructed from concatenated SNVs. Metagenome-derived hospital strains from this study demarcated as the purple clade. ANI comparisons and a *C. albicans* SNV tree are also available in Figures S1–S2. **B** Whole genome SNV density plots for each *C. parapsilosis* strain. Strain names in red are strains assembled from samples from infants or the NICU from Magee-Women’s Hospital. SNV density plotted in 1.3 kb sliding windows. Window size was selected based on ease of visualization. Chromosomes are separated with dashed lines. Total bar height represents total SNV density, and homozygous SNV proportion is labeled in red whereas heterozygous is black. **C** Depiction of SNV hotspot overlaps between each strain. Pairwise overlap was calculated between each strain and plotted. Strain names in red are strains assembled from samples from infants or the NICU from Magee-Women’s Hospital. **D** Two example SNV hotspots. Individual SNVs are represented with red bars

Because multiple new strains were sequenced from the same hospital, the phylogenetic relationships of new and previously sequenced strains from the same hospital were of interest from the perspectives of the persistence of Candida populations in the hospital environment and transfer from room to human. To place the hospital and gut-associated sequences in context, we first compared those genomes to available reference genomes from NCBI using pair-wise average nucleotide identity (ANI) and by construction of single nucleotide variant (SNV) trees (Fig. 1A, Figure S1–S2). L2_023 was not included due to low sequencing coverage. *C. albicans* strains were spread throughout the tree of known *C. albicans* diversity (Figure S2) whereas *C. parapsilosis* strains from infant gut and NICU samples were clustered on a single branch (Fig. 1A) separate from other reference hospital and environmental strains. Further, the two infant gut strains, sampled years apart (Table 1), were nearly identical (99.99% identity). We verified this with whole genome alignments of the hospital and gut sequences (Figure S1–S2). We thus infer that the hospital room and gut *C. parapsilosis* strains are very closely related and are indicative of a possible hospital-associated *C. parapsilosis* strain sequenced multiple times, years apart.

Based on analysis of population structure of all seven unique *C. parapsilosis* genomes (Figure S3), we predicted six distinct *C. parapsilosis* ancestral populations. The exception is the fecal strain N3_182, which appears to be a recombinant admixture of the ancestral populations NICU strain S2_005 and the fecal strain C1_006. Given that N3_182 was sequenced 4 years before C1_006 (Table 1), both parental strains may have existed in the hospital environment prior to hybridization. The findings provide further evidence of distinct hospital-associated *C. parapsilosis* strains, a hybrid of which colonized a premature infant. However, it may be difficult to accurately determine fine-grained population structure with small genome sample sizes, and future sequencing of *C. parapsilosis* genomes may further clarify this result.

### C. parapsilosis SNV hotspots as indicators of genes under selection

To investigate whether genomes sampled from the hospital could provide evidence of evolutionary adaptation to this environment, we visualized the spatial distribution of *C. parapsilosis* genomic diversity in the newly reconstructed genomes by mapping reads from each genome to a reference sequence (CDC317, recovered from a clinical sample) and calling SNVs. We plotted the density of SNVs in 1.3 kbp sliding windows across the genome of each strain (Fig. 1B). Both heterozygous and homozygous SNVs are largely evenly distributed throughout the genome, with the exception of a few small regions with highly elevated SNV counts (regions of elevated diversity) that we refer to as SNV hotspots (Fig. 1B).

Interestingly, SNV hotspots show a high level of conservation between all strains (Fig. 1C). The one exception is reference strain GA1 cultured from human blood [23], which shares only ~ 10% of its SNV hotspots with any other given strain. Notably, the NYC subway strain is fairly similar to the clinical reference strain CDC317 used for mapping (few and minor hotspots) whereas our hospital sequences share SNV hotspots with environmental reference strains CBS1954 and CBS6318 (one isolated from an olive and the other from healthy human skin).

To provide a more complete view of SNV hotspots and ensure they were not an artifact of SNV hotspots solely present in the CDC317 reference genome, we also mapped the reads from each population to three other genomes (environmental strains CBS1984 and CBS6318, and the GA1 blood isolate, Figure S4). The number of SNV hotspots ranged from 16 to 45, and the regions were 5 kb to 24.5 kb in length. Due to the large size of the SNV hotspots, each hotspot overlaps a number of individual genes with SNVs spread both within and between genes (Fig. 1D). In total, 376 genes are present within a SNV hotspot in at least one strain. No particular KEGG family or PFAM domain was significantly enriched in SNV hotspots.

### Multicopy RTA3 gene

**Fig. 2:**
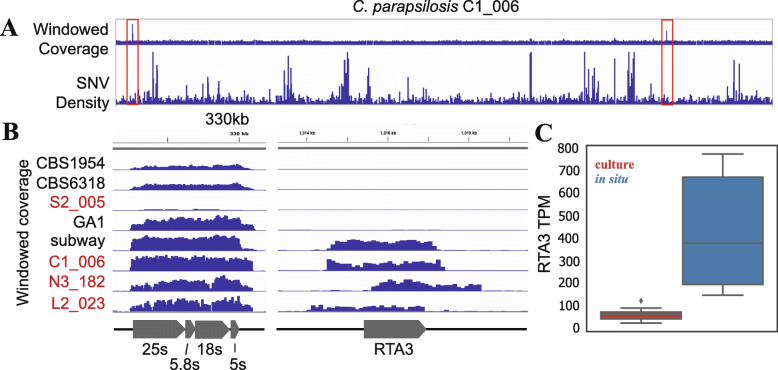
*C. parapsilosis* strains have high copy number rRNA and RTA3 loci. **A** Whole genome windowed coverage of SNP density for *C. parapsilosis* strain C1_006. High copy number regions of interest are highlighted with red boxes. **B** An expanded view of highlighted high copy number regions from **A**. Windowed coverage is plotted as 100 bp sliding windows. Metagenome-derived hospital strains from this study labeled in red. **C** Boxplot of expression of the RTA3 gene from multicopy strain C1_006 in situ (blue) and strain CDC317 in culture (red). Expression represented as transcripts per million (TPM). Expression is significantly different between the two groupings (*p* = 0.004) as determined using the Wilcoxon ranked-sums test

Another explanation for SNV hotspots could be due to gene copy number variation, as recent duplications of a region acquire mutations yet reads from these duplications map back to a single location. Overall, when windowed genomic coverage is plotted alongside SNV density (Fig. 2A), this is clearly not the case. However, across the entire genome two regions of high coverage (Fig. 2A), indicating high copy number variation, were identified and neither correspond to SNV hotspots. The first high copy number region contains an estimated 17–28 copies of the 18S, 25S, 5S, and 5.5S rRNA genes (Table S1, Fig. 2B). The variation in rRNA copy number may indicate a range of maximum growth rates [25]. The second region, which corresponds to the lipid translocase RTA3 gene and flanking sequence, is present in 5–16 copies (Table S1) in strains C1_006, N3_182, L2_023, S2_005, and NYC_subway but is single-copy in the four isolate genomes (Fig. 2B). Interestingly, RTA3 has been implicated in resistance to azole class antifungal drugs such as fluconazole in *C. albicans* [19]. The high copy number RTA3 genes also have no detectable SNVs and different boundaries in each strain, suggesting the duplications may be recent and independent events in each strain.

### In situ metatranscriptomics and metaproteomics

**Fig. 3:**
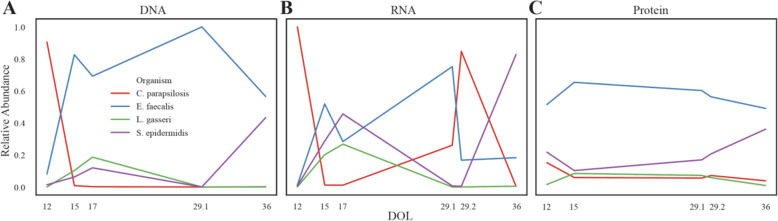
In situ metagenomics metatranscriptomics, and metaproteomics of infant 06. Plotted are the relative DNA, RNA, and peptide abundances for each detected organism after human removal. Plotted on the x axis are the days of life (DOL) samples that were taken

Given most work with Candida species is performed in pure culture or in murine models, little is known about their behavior in the human gut. We hypothesized performing metatranscriptomics and metaproteomics on infant fecal samples with Candida would reveal unique transcriptomic and proteomic profiles, indicative of differences in metabolism and behavior between culture and in situ settings. Two candidate infants were identified: infant 06 with a documented Candida blood infection (Fig. 3) and infant 74 with a documented Candida lung infection. Both infants were treated with fluconazole shortly after detection of Candida infection (Fig. 3, Table S2). Metagenomic, metatranscriptomic, and metaproteomic datasets were generated from fecal samples at five to six timepoints for each infant. In infant 74, no Candida species were detected in the generated datasets (Figure S4). However, in infant 06, metagenomic sequencing confirmed the presence of *C. parapsilosis* (strain C1_006) in the fecal samples. De novo gene prediction was performed on the metagenome-derived *C. parapsilosis* genome and the resulting gene models were used for mapping transcriptomic reads and proteomic peptides (Fig. 3).

In addition to *C. parapsilosis*, genomes were recovered for three bacterial species in infant 06: *Enterococcus faecalis*, *Lactobacillus gasseri*, and *Staphylococcus epidermidis*. It is not uncommon for only four organisms, or even fewer, to be present within a premature infant gut metagenome as the infant gut is normally sterile at birth and premature infants in particular typically receive antibiotics in the first weeks of life [21]. Interestingly, in every infant where a Candida genome was assembled or detected through read mapping, *E. faecalis* was also present (*N* = 7). *C. parapsilosis* is highly abundant relative to other organisms in the first 20 days of life before quickly being replaced or outnumbered, largely by *E. faecalis*. Similar abundance patterns have been observed previously for microbial eukaryotes in neonatal fecal samples [18]. *C. parapsilosis* transcriptomic abundance shows a similar pattern to the DNA abundance but transcription remains detectable at later time points (Fig. 3). In contrast, *C. parapsilosis* proteomic abundance remained relatively stable over all timepoints.

### C. parapsilosis expression in situ vs. culture settings

**Fig. 4:**
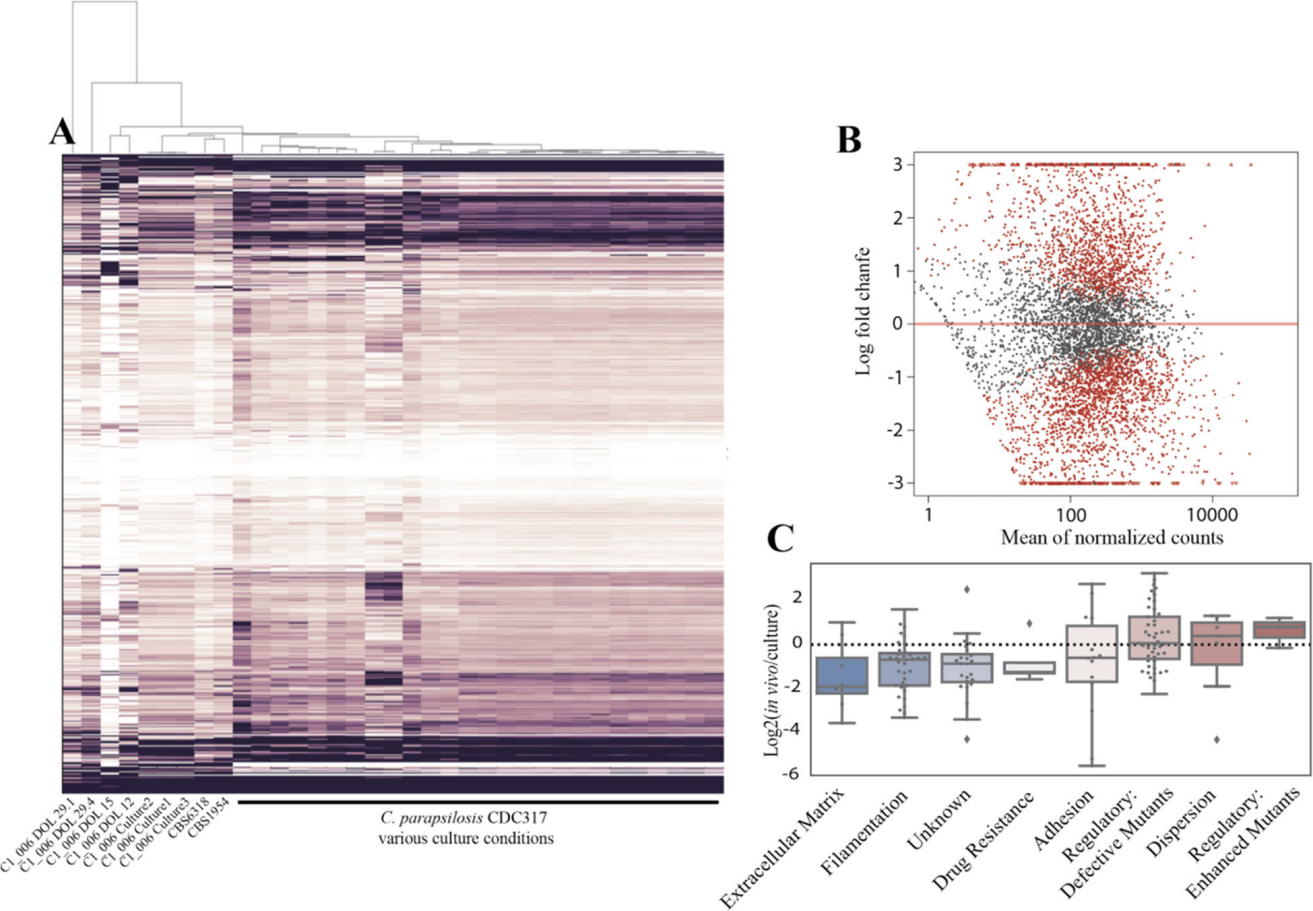
*C. parapsilosis* displays distinct and highly variable in situ transcriptomic profiles. **A** Hierarchical clustering of *C. parapsilosis* TPM values for C1_006 in in situ samples and pure culture samples under a variety of conditions. **B** Average log2 fold change in situ vs culture plotted against the mean of normalized counts for each transcript. Transcripts in red were identified as being significantly differentially expressed by DESeq2. **C** Boxplots of expression of categories of genes involved in biofilm formation. Gene lists and categories were obtained from [27]. Regulatory defective mutants refers to regulatory genes that inhibit biofilm formation when mutated, regulatory enhanced refer to genes that increase biofilm formation when mutated, and unknown refers to genes involved in biofilm formation but their exact role is unclear

Given most work with *C. parapsilosis* has been performed on pure cultures, we wondered if there are differences in behavior and metabolism in situ that would be detectable by comparing transcriptomic datasets. For comparison, *C. parapsilosis* strain C1_06 was isolated from infant 06 fecal material on DOL 12. Transcriptomic datasets were then generated for cultures of the C1_06 isolate grown in YPD at 30 °C to replicate standard Candida isolate culture conditions. In addition, we downloaded raw sequencing reads from publicly available *C. parapsilosis* RNAseq experiments [23, 26], including datasets from multiple strains (CDC317, CBS1954, and CBS6318) and varying culture conditions, including different media, growth temperatures, and oxygen concentrations. A hierarchical clustering of expression of CDC317 transcripts reveals a clearly distinct transcriptomic profile between in situ and all culture settings (Fig. 4A). Importantly, C1_06 culture transcriptomes cluster closer to culture transcriptomes of various other strains than to C1_06 in situ samples. Notably, in situ samples are also extremely variable; clustering as far apart from one another as from the culture samples (Fig. 4A). We quantitatively identified differentially expressed transcripts between culture and in situ settings with DESeq2 and found that 53% of transcripts were significantly differentially expressed; 23% up in situ, 30% down (Fig. 4B), further highlighting the stark differences between in situ and culture settings.

Biofilm formation is an important virulence factor for Candida species, often contributing to the development of systemic infections [27, 28]. We were interested in whether the expression of virulence factors was enriched in situ, given the samples were obtained from an infant with a documented Candida blood infection. We obtained a list of well-characterized biofilm formation genes from *C. albicans* [27], identified orthologs in *C. parapsilosis* and compared their expression in situ to culture settings*.* Biofilm formation showed lower expression overall in situ (Fig. 4C).

**Fig. 5:**
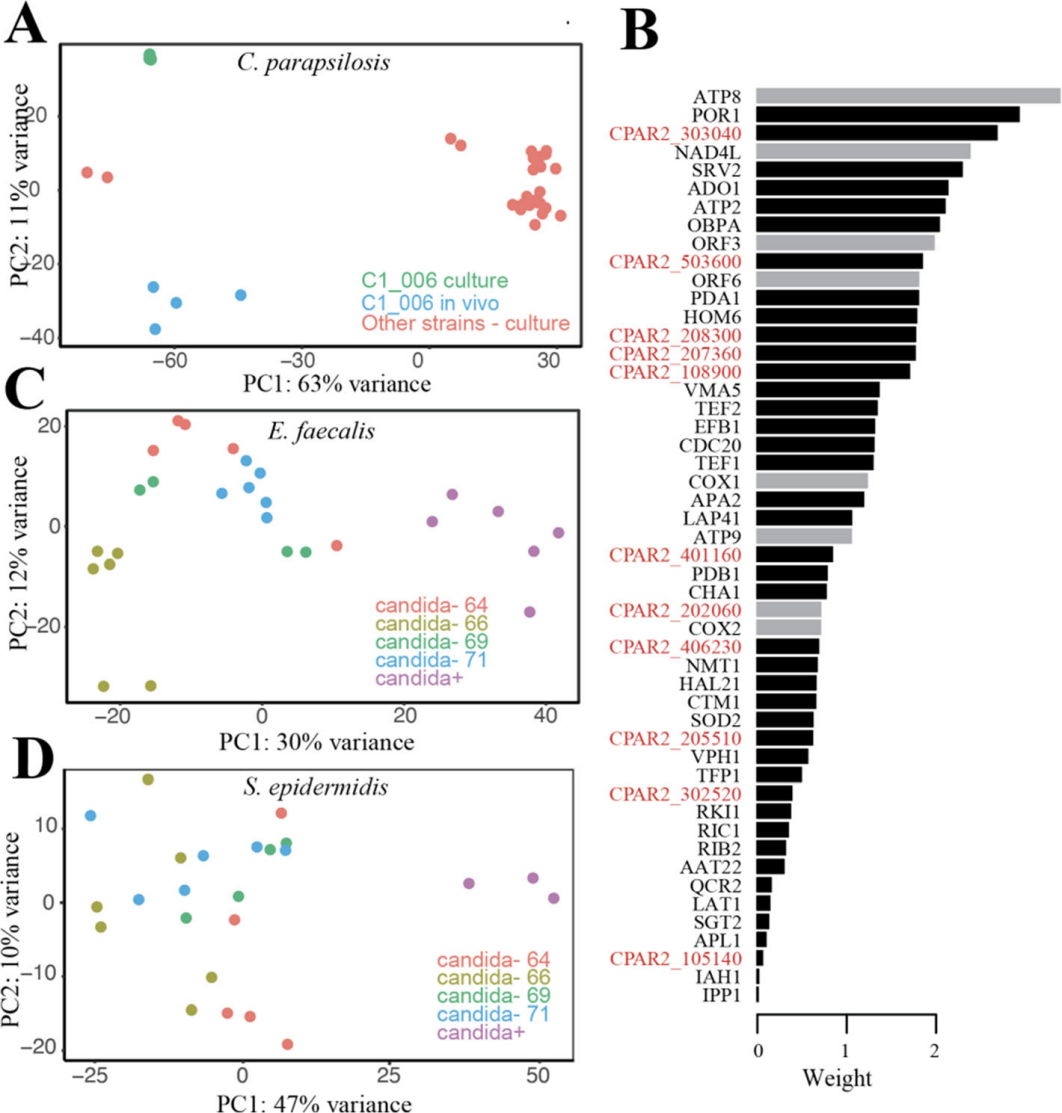
Presence of *C. parapsilosis* affects bacterial community member’s expression. **A** PCA of *C. parapsilosis* in situ and pure culture transcriptomes. **B** Depiction of features identified by sPLS-DA for separating *C. parapsilosis in situ* and pure culture transcriptomes. Plotted are the feature weights. Black bars are genes that exhibited higher expression on average in situ whereas grey had higher average expression in culture. Genes labeled in red correspond to proteins of unknown function. **C**, **D** PCAs of *E faecalis* (**C**) and *S. epidermidis* (**D**) transcriptomes from infant microbiomes both with and without detected *C. parapsilosis*. Candida-negative transcriptomes were from four different infants (published previously; Sher et al. 2020) denoted as 64, 66, 69, and 71

In situ and culture transcriptome samples were differentiable in a principal component analysis (PCA), paralleling the hierarchical clustering of *C. parapsilosis* transcriptomes (Fig. 5A), although C1_006 culture transcriptomes did not cluster as closely to other strain culture samples in this analysis. We performed a sparse partial least squares discriminant analysis (sPLS-DA), treating each transcript as a variable, to try and identify important features able to discriminate between in situ and culture in a multivariate space (Fig. 5B, Figure S5, Table S3). Important features were enriched for mitochondrial and aerobic respiration genes (9/50) and uncharacterized genes (11/50).

We were curious to see if the multicopy RTA3 gene in infant strain C1_006 (Fig. 2B) showed increased expression as compared to the single copy RTA3 gene in reference strain CDC317. Indeed, the expression of the RTA3 in strain C1_006 is significantly higher (*p* = 0.004, Fig. 2C), suggesting a role of this gene duplication as a way to increase overall expression of RTA3. Interestingly, there was no significant difference in strain C1_006 RTA3 expression between culture and in situ settings (Figure S6A), and we did not see an increase in expression following fluconazole treatment *in situ* (Figure S6B), indicating RTA3 expression may be constitutively higher in C1_006 regardless of condition. However, it is worth noting we were unable to obtain samples until 7 days after fluconazole treatment and any treatment effect on expression may have already passed.

### C. parapsilosis impact on bacterial expression

*E. faecalis*, *S. epidermidis*, and *L. gasseri* bacteria in infant 06 had transcripts sequenced at high depths at multiple time points (Fig. 3). So, it was possible to investigate whether the presence or absence of *C. parapsilosis* had a distinguishable effect on their transcriptomic profiles. We compared bacterial transcription in these samples to transcription patterns of bacteria in the absence of Candida using previously reported datasets (21 samples for *E. faecalis* and 20 samples for *S. epidermidis* [29]). The analysis was not possible for *L. gasseri* as this bacterium was not present in any of the metatranscriptomes used for comparison. The transcriptomes of *S. epidermidis* were distinguishable between the presence and absence of *C. parapsilosis*, and this effect appears to be independent of infant of origin and thus the bacterial strain variant type (Fig. 5C, D). The effect is also present for *E. faecalis*, although less clear and could possibly be explained by variance across infants. This result suggests *C. parapsilosis* has a large impact on the behavior and metabolism of other gut community members. In addition, the expression of *E. faecalis* genes previously shown to negatively impact *C. albicans* virulence [15] showed no significant difference in expression between *C. parapsilosi*s negative and positive samples.

Important features identified from a sPLS-DA on Candida-positive vs. Candida-negative samples included a subset of *E. faecalis* ribosomal proteins (Table S3, Figure S5). Additionally, ribosomal proteins all showed higher expression in situ, suggesting increased *E. faecalis* growth rate in the presence of *C. parapsilosis*. Other important features included mannitol-specific phosphotransferase system (PTS) transporters upregulated in Candida-positive samples and downregulated mannose-specific PTS transporters (Table S3). Furthermore, Mannitol-1-phosphate 5-dehydrogenase, an enzyme responsible for the conversion of D-mannitol to fructose, was upregulated in Candida-positive samples, indicating an increased capacity for degradation of mannitol in addition to import (Table S3). Important features in *S. epidermidis* were less clear, but again included a subset of ribosomal proteins as well as beta-lactamases, both with increased expression in situ (Table S3).

### Transcriptomics enriched gene functions

**Fig. 6:**
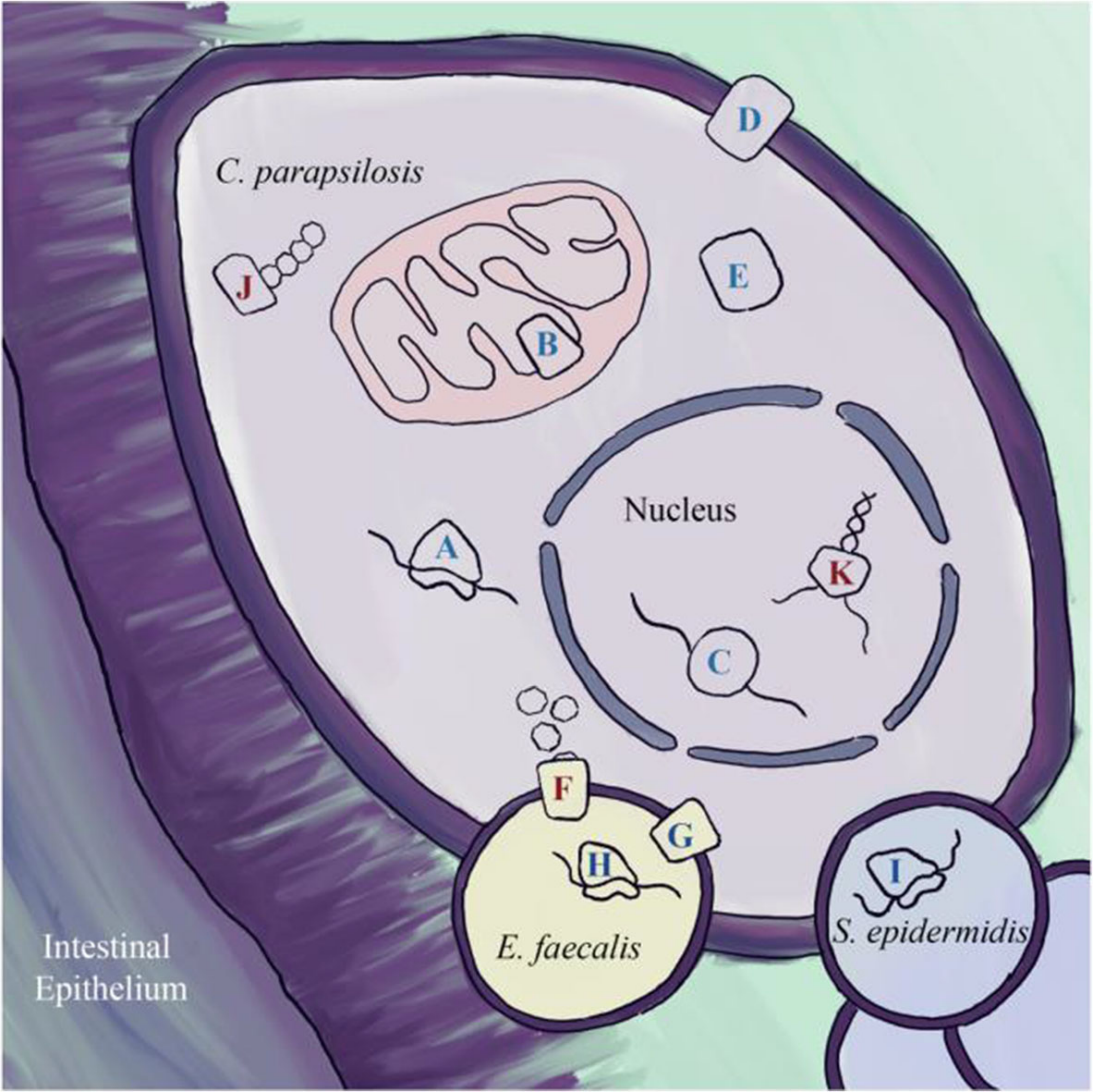
In situ enriched gene categories. Diagram depicting *C. parapsilosis* in the context of the infant gut, highlighting gene categories or families that were significantly enriched in differentially expressed genes between in situ and culture. Blue letters represent functions with higher expression in situ, while red represent functions with lower expression in situ. See Table S5 for details. **A** Ribosomal proteins, **B** cytochrome c oxidase subunits, **C** LSM complexes, **D** proton antiporters, **F** *E. faecalis* mannose transporters, **G** *E. faecalis* mannitol transporters, **H** *E. faecalis* subset of ribosomal proteins, **I** *S. epidermidis* subset of ribosomal proteins, **J** *C. parapsilosis* polysaccharide synthases (downregulated in situ), **K** *C. parapsilosis* helicases (downregulated in situ).

Given the large differences in transcriptomes between culture and in situ *C. parapsilosis*, we looked for functions enriched in either setting (Fig. 6, Table S4). DESeq2 identified groups of differentially expressed genes that were too large to be informative, so more restrictive cutoffs were used. Up in situ was defined as having > 3 log2 expression in situ whereas down in situ was defined as < − 3 log2 expression in situ. Up in situ was enriched for KEGG families for LSM 2–8 and 1–7 complexes, a family of proteins involved in mRNA metabolism highly conserved in eukaryotes [30], as well as Cytochrome c oxidase and bc1 complex and proteins without an annotated KEGG family (Fig. 6, Table S4). Down in situ is enriched for helicase and polysaccharide synthase PFAM domains. Additionally, proteins without an annotated KEGG family (unknown function) were enriched in both groups (Table S4).

### Proteomics

Across the 10 proteome samples from 5 timepoints in infant 06, 7063 protein groups (groups of similar sequences that cannot be distinguished because the peptides are shared) were quantified, with an average of 4872 protein groups at each time point. Among these were 5312 human and 1751 protein groups from *C. parapsilosis* and bacteria (Supplemental Figure S7). Human protein groups dominate (90% of the peptide abundances in each sample) because the limited amount of fecal material precluded depletion of human cells before cellular lysis and protein extraction. Among the quantified human protein groups, 324 of the 480 involved in neutrophil degranulation (67%) were identified, with an average of 294 protein groups detected at each sampling point. This indicates an active host immune response [31] (Supplemental Table S5).

While the high representation of human protein groups reduces the coverage depth of the microbial membership, it allows for simultaneous examination of both host and microbiome activities. We quantified 349 *C. parapsilosis* protein groups across all samples measured, with a minimum of 126 *C. parapsilosis* protein groups per sample. While this represents only ~ 6% of the predicted *C. parapsilosis* proteome, the data enabled observation of stability across time and determination of some of the general metabolic activities of this organism (Supplemental Figure S8). We detected evidence for *C. parapsilosis* core metabolic activities such as glycolysis, the TCA cycle, and organic acid metabolism. Repeated detection of similar abundances of these proteins across the 24-day timespan of collected samples indicates the stability of *C. parapsilosis* in the gut environment.

The abundant, significantly enriched protein groups from *C. parapsilosis* were ribosomal and F-Type ATPase proteins (Fig. 6), HSP70 and proteins with actin PFAM domains (Table S4). Protein groups with the most peptide evidence were involved in protection from oxidative stress (e.g., superoxide dismutase). This suggests that *C. parapsilosis* was actively responding to, and adapting to, environmental stressors. Included in the set of highly abundant proteins were some encoded in genome regions within SNV hotspots. However, we found no significant association between protein abundance and association with these hotspots. We also examined the most abundant proteins in the bacterial species. In *E. faecalis* and *S. epidermidis*, proteins of the Lac pathway were among the most abundant bacterial proteins. This suggests that lactose may be an important substrate for these community members in situ.

## Discussion

Fungal pathogens are known to have hospital reservoirs. For example, the water supply system of a pediatric institute was shown to be a reservoir for *Fusarium solani* [32]. A NICU outbreak of the fungi *Malassezia pachydermatis* was linked to the dog of a healthcare worker [33], although persistence via long-term carriage by a healthcare worker vs. continual passage between infants and rooms (or a combination of these) could not be resolved. However, much remains to be learned about where reservoirs of hospital-associated fungi are and how long strains persist in them. In contrast to previous studies of *C. parapsilosis* utilizing pure culture and model systems, we applied genome-resolved metagenomics, metatranscriptomics, and metaproteomics to study *C. parapsilosis* in the context of the infant gut and hospital rooms of a neonatal intensive care unit. We detected novel, near identical *C. parapsilosis* genomes sequenced years apart in separate infants, suggesting transmission of members of a fungal population from reservoir to infant or infant to reservoir to infant. It is worth noting that although the strains are near-identical, the multicopy RTA3 locus in each strain had different boundaries and different copy numbers. This observation suggests that these two strains are very closely related members of a somewhat more diverse hospital adapted population.

Population genomic analyses of reconstructed genomes revealed multiple, independent instances of copy number gain of the RTA3 gene. RTA3, a lipid translocase, has been implicated in resistance to azole class antifungal drugs such as fluconazole in *C. albicans* [19]. The RTA3 gene is frequently overexpressed in resistant isolates and increased expression of RTA3 increases resistance to fluconazole whereas deletion of the RTA3 gene results in increased azole susceptibility [19]. Copy gain of this gene in *C. parapsilosis* strains may represent a mechanism for rapid adaptation to fluconazole, the most widely used antifungal in most hospitals [19], as a means by which to increase its expression and thus its resistance. Similar gene copy number gains have been reported for the human amylase gene, hypothesized to be in response to increases in starch consumption [34]. Indeed, RTA3 expression in situ from strain C1_006, which has RTA3 in multicopy, was significantly increased as compared to single copy strain CDC317 in culture [26] (Fig. 2C). The high likelihood that the copy number gain occurred independently in multiple strains suggests selection for this particular genomic feature. Identifying mechanisms of antifungal resistance is of particular importance given 3–5% of *C. parapsilosis* strains are already resistant to fluconazole [10] and our relative inability to deal with infections of drug-resistant fungi.

Examining the genomic distribution of SNVs within the genomes of each *C. parapsilosis* strain revealed the presence of SNV hotspots. Interestingly, no particular KEGG family or PFAM domain was significantly enriched within SNV hotspots. This, combined with the fact that SNVs within hotspots are spread both within and between genes, may be indicative of the identified SNV hotspots being recombination hotspots, or locations where many additional SNVs hitchhike along with SNVs under selection as many of these SNVs, particularly those in non-coding regions, are likely to have little to no effect on function.

Many of these SNV hotspots are shared between strains, some of which are specific to the hospital and infant gut strains. Unlike *C. albicans*, *C. parapsilosis* is not an obligate commensal of mammals [6]. Consequently, some regions of the *C. parapsilosis* genome may be under selection for adaptation to the hospital, in addition to the gut environment. Further supporting the idea that some genomic innovation is associated with adaptation to the built environment, the NICU strain clustered the most closely to the NYC subway strain based on SNV hotspot overlap (Fig. 1C). These two strains are geographically and phylogenetically distinct, but the shared regions of diversification may be related to their common need to adapt to the built environment.

Metatranscriptomics of infant fecal samples revealed *C. parapsilosis* transcriptomes that are both highly variable and distinct from those of culture samples. Interestingly, the degree of variance exhibited by transcriptomes of the same population in the same infant over a few day period was greater than that observed between *C. albicans* white and opaque phenotypes (Figure S9) [35]. The *C. albicans* white and opaque phenotypes differ in their appearance [36], mating style [37], and environmental conditions they are best adapted to [38, 39], and represent two exceptionally distinct Candida phenotypes. The high variability in *C. parapsilosis* is likely the result of changing conditions presented in the gut, including microbial community composition as well as the developing physiology of the host. Varying stages of infection and/or response to antifungal treatment may also have had an effect, but more dense time-series and additional infants would be required to elucidate these effects.

In contrast to the large changes in *C. parapsilosis* RNA and DNA relative abundances over time, *C. parapsilosis* peptide relative abundance remained stable over the study period. It is not uncommon to see different signals from transcripts and proteins [40], in part because proteins can persist for relatively long periods of time compared to transcripts. The most abundant proteins in the proteomics dataset have a HSP70 domain found in heat shock proteins (HSP). In *C. albicans*, HSP have been documented to help control virulence by interacting with regulatory systems and to enable drug resistance [41].

The presence of *C. parapsilosis* within infant gut samples may impact the transcriptomes of bacterial gut community members. Important features for separating Candida-positive and Candida-negative *E. faecalis* transcriptomic samples included a suite of upregulated mannitol transporters and downregulated mannose transporters (Table S3). *C. parapsilosis* strain SK26.001 is documented as producing mannitol [42] and mannose, in the form of the polysaccharide mannan which can be an important component of extracellular polysaccharides produced by Candida [43]. Interestingly, a characteristic of *E. faecalis* is its ability to grow by fermenting mannitol [44]. Given the potential for interaction between *E. faecalis* and *C. parapsilosis*, it is possible that the presence of *C. parapsilosis* induces a substrate switch in *E. faecalis* from mannose, an important component of *C. parapsilosis* biofilm matrix, to mannitol, a sugar produced by *C. parapsilosis* under some conditions.

Interestingly, statistical tests detected a subset of ribosomal proteins as important features for separating Candida-positive from Candida-negative samples for both *E. faecalis* and *S. epidermidis* (Table S3) based on transcription. In recent years, ribosomal heterogeneity, in which ribosomal protein subunits are swapped out or missing from individual ribosomes, has gained traction as a way for organisms to regulate translation [45–47]. Ribosomal heterogeneity may be being utilized as an additional regulatory measure to adapt to the rapidly changing gut microbial context. Alternatively, fluctuations in ribosomal subunit abundance could be to maintain ribosomal homeostasis [48], or individual ribosomal subunits could be performing functions unrelated to protein synthesis [49].

Biofilm formation is an important virulence factor of Candida infections [50]. Infant 06 had a documented Candida blood infection, and such infections are commonly systemic [51]. Interestingly, despite infection, Candida biofilm formation genes were relatively less expressed in situ in the gut of Infant 06 as compared to expression levels previously reported over a range of culture conditions. Similarly, genes with a PFAM domain for polysaccharide synthase, genes potentially important for the generation of Candida biofilm matrices [43], were less expressed in situ than in cultures. Thus, biofilm formation may not be an important component of every infection.

The prevalence of transcripts of uncharacterized genes in the in situ transcriptomes (Fig. 5B; Table S3) is particularly interesting. *C. parapsilosis* and other Candida species are rarely studied in a microbial community context, leaving gaps in understanding of genes required for organism-organism interactions. We suspect that some of the highly expressed genes are important for Candida interactions with bacteria and other community members. Thus, they represent important targets for future co-culture-based investigations.

A limitation of this study was obtaining fecal samples with sufficient Candida DNA, RNA, and protein for analysis. Consequently, although we present the first in situ metatranscriptomics and metaproteomics for *C. parapsilosis*, the data analyzed is for a single strain in a single infant. It is perhaps not unexpected that in situ expression patterns differed significantly from those observed in culture settings. However, metatransciptomes from more *C. parapsilosis* strains and more infants that are recovered under highly standardized conditions are needed to determine the contributing factors, such as the coexisting bacteria and infant gut conditions that lead to these differences. Development of methods to more reliably recover low abundance microbial eukaryotic material in the midst of the bacterially dominated gut will be crucial for further insights.

## Conclusions

We applied genome-resolved metagenomics, metatranscriptomics, and metaproteomics to recover genomes for, and study the behavior of, *C. parapsilosis* in situ. We showed *C. parapsilosis* has a highly distinct transcriptomic profile in situ vs in culture. Further, the extreme variability in the in situ transcriptome data indicates the considerable effect the gut microbial community and human host may have on *C. parapsilosis* behavior and metabolism. Overall, these results demonstrate that in situ study of *C. parapsilosis* and other Candida species is not only possible but necessary for a more holistic understanding of their biology.

## Methods

### Metagenomic sampling and sequencing

All infant fecal metagenomes used in this study were derived from infants housed in the Magee-Womens Hospital (Pittsburgh, PA). This study made use of previously published infant datasets: NIH1 [52], NIH2 [53], NIH3 [54], NIH4 [55], Sloan2 [53], and SP_CRL [56], as well as several new datasets including multiple timepoints from infant 06 and infant 74, and samples L2_023, S3_003, and S3_016.

For newly generated metagenomic sequencing from infant 06 and infant 74, total genomic DNA and total RNA were extracted from previously unanalyzed fecal samples using Qiagen’s AllPrep PowerFecal DNA/RNA kit (Qiagen) and subsequently split into DNA and RNA portions. The aliquot used for metagenomic sample preparation was treated with RNase A. DNA quality and concentration were verified with Qubit (Thermofisher) and Fragment Analyzer (Agilent). Illumina libraries with an average insert size of 300 bps were constructed from purified genomic DNA using the Nextera XT kit (Illumina) and sequenced on Illumina’s NovaSeq platform in a paired end 140 bp read configuration, resulting in at least 130 million paired end reads from each library.

NICU metagenomic sampling was described and published previously [53]. All samples were collected from the same NICU at UPMC Magee-Womens Hospital (Pittsburgh, PA). In order to generate enough DNA for metagenomic sequencing, DNA was collected from multiple sites in the NICU and combined into three separate pools for sequencing. Highly touched surfaces included samples originating from the isolette handrail, isolette knobs, nurses hands, in-room phone, chair armrest, computer mouse, computer monitor, and computer keyboard. Sink samples included samples from the bottom of the sink basin and drain. Counters and floors consisted of the room floor and surface of the isolette. See previous publication for details [53, 57].

### Eukaryotic genome binning and gene prediction

For each sample, sequencing reads were assembled independently with IDBA-UD [58]. Additionally, for each infant, reads from every time point were concatenated together. A co-assembly was then performed on the pooled reads for each infant with IDBA-UD in order to assemble sequences from low abundance organisms. The Eukaryotic porton of each sample assembly was predicted with EukRep [17] and putative eukaryotic bins were generated by running CONCOCT [59] with default settings on the output of EukRep. To reduce computational load, resulting eukaryotic bins shorter than 2.5 mbp in length were not included in further analyses. GeneMark-ES [60] and AUGUSTUS [61] trained with BUSCO [62] were used to perform gene prediction on each bin using the MAKER [63] pipeline. In addition, a second homology-based gene prediction step was performed. Each bin was identified as either *C. parapsilosis* or *C. albicans* and reference gene sets from *C. parapsilosis CDC317* and *C. albicans SC5314* were used for homology evidence respectively in a second-pass gene prediction step with AUGUSTUS [61], as implemented in MAKER [63].

### Bacterial genome binning and gene prediction

For infant 06 and infant 74 metagenomes, bacterial genes were predicted on whole metagenomes using Prodigal in metagenome mode (-p meta option; Hyatt et al. 2012). Predicted proteins were functionally and taxonomically annotated by searching against Uniprot (The UniProt Consortium 2017), KEGG (Kanehisa et al. 2016), and Uniref90 (Suzek et al. 2007) with USEARCH (UBLAST) (Edgar 2010). Taxonomy for scaffolds was then determined by taking the consensus of closest hits of each individual gene sequence on a contig and determining the winner by majority. Bacterial genomes were then manually binned with ggKbase (ggkbase.berkeley.edu) utilizing coverage, GC, and taxonomic information.

### SNV calling and detection of SNV hotspots

In order to call variants in each Candida genome, reads from the sample in which a particular genome was binned from, or the publically available reads from SRA, were mapped back to the de novo assembled genome using Bowtie 2 [64] with default parameters. The PicardTool (http://broadinstitute.github.io/picard/) functions “SortSam” and “MarkDuplicates” were used to sort the resulting sam file and remove duplicate reads. FreeBayes (Garrison et al. 2012) was used to perform variant calling with the options “--pooled-continuous -F 0.01 -C 1.” Variants were filtered downstream to include only those with support of at least 10% of total mapped reads in order to avoid false positives. SNV read counts were calculated using the “AO” and “RO” fields in the FreeBayes vcf output file.

SNV density was visualized across the CDC317 reference genome using a custom python script (https://github.com/patrickwest/c_parapsilosis_analysis). SNV hotspots were quantitatively defined with 5 kbp windows with a slide of 500 bp across the genome, flagging windows with a SNV density at least three standard deviations above the genomic average SNV density, and merging overlapping flagged windows. Genes located within SNV hotspots as well as overlapping SNV hotspots between strains were identified with intersectBed [65].

### Candida phylogenetics and population structure

For generation of a SNP tree for both *C. parapsilosis* and *C. albicans*, all publically available genomic sequencing reads for both species were downloaded from NCBI’s short read archive (SRA), including 4 isolate *C. parapsilosis* read sets and 51 *C. albicans* sets. SNVs were called for each isolate read set using the same pipeline used for metagenome-derived genomes, as described above. A SNP tree was generated for *C. parapsilosis* and *C. albicans* using SNPhylo [66] with settings ‘-r -M 0.5 -l 2’ and ‘-r -M 0.5 -l 0.8’ respectively and visualized using FigTree (https://github.com/rambaut/figtree/). For genomic average nucleotide identity (ANI) comparisons, 4 *C. parapsilosis* and 51 *C. albicans* reference genomes were downloaded from NCBI. Subsequently, dRep [67] in the ‘compare_wf’ setting was used to generate ANI comparisons for each genome. For inferring *C. parapsilosis* population structure, FreeBayes vcf files were converted to PLINK bed format with PLINK [68] and used as input for ADMIXTURE [69]. A total of 3785 SNVs were used to infer ancestral populations. The predicted number of ancestral populations, K, was selected using ADMIXTURE’s cross-validation procedure for values 1–8.

### Detection of copy number variation

Genomic copy number variation within the *C. parapsilosis* strains was searched for by mapping reads from the sample the genome was derived from to the *C. parapsilosis* CDC317 reference genome. Windowed coverage was then calculated across the genome in 100 bp sliding windows using pipeCoverage (https://github.com/MrOlm/pipeCoverage) and visualized with Integrated Genomics Viewer (IGV) [70]. Copy numbers for multicopy regions were estimated by dividing the average coverage of the windows located within the multicopy region by the average genomic coverage.

### Transcriptomic sequencing and analysis

For in vivo metatranscriptome generation, total RNA was extracted from fecal samples using the AllPrep PowerFecal DNA/RNA kit (Qiagen) and subsequently treated with DNase. Purified RNA quality and concentration were measured using the Fragment Analyzer (Agilent). Illumina sequencing libraries were constructed with the ScriptSeq Complete Gold Kit (Illumina) without performing the rRNA removal step, resulting in library molecules with an average insert size of 150 bp. Sequencing was performed on Illumina’s NextSeq platform in a paired end 75 bp configuration, resulting in an average of 54 million paired end reads per sample.

For culture *C. parapsilosis* strain C1_06 transcriptomes, strain C1_06 was isolated from the stool of Infant patient 06 on day 12 of life. Cultures of this isolate were grown in YPD at 30 °C to mid-log phase. All cultures were pelleted, washed, and flash frozen in liquid nitrogen. RNA was extracted using the RiboPure RNA Purification kit (Ambion) and RNA samples were submitted to the JP Sulzberger Columbia Genome Center for library preparation and sequencing. Libraries were constructed using the Illumina TruSeq RNA library prep kit v2 and 100 bp single-end reads were sequenced using the Illumina NovaSeq.

Transcriptomic reads from studies [23, 26] were downloaded from the SRA. Transcriptomic reads from each dataset were then mapped to *C. parapsilosis* reference strain CDC317 gene models with Kallisto [71] and transcript per million (TPM) values were used to compare expression levels across samples. Differentially expressed transcripts were identified using raw read counts with the R package DESeq2 [72]. Rlog transformation was applied to transcript read counts from each sample prior to generation of transcriptome PCAs. PCA plots were generated with DESeq2. Important features for separating C. parapsilosis in situ and culture as well as *E. faecalis* and *S. epidermidis* Candida-positive and Candida-negative samples were identified through the use of a sparse Partial Least Squares Discriminant Analysis (sPLS-DA) as implemented in the MixOmics package [73] on rlog transformed transcript read counts. MixOmics cross-validation (tune.splsda) was used with settings fold = 3 and nrepeat =50 to estimate the optimal number of components (features) for separating each pair of sample types.

Genes were annotated with KEGG KOs and PFAM domains using HMMER with KOfam [74] and Pfam-A [75] HMM databases. Subsets of genes of interest (described in results) were then searched for significantly enriched KEGG families or PFAM domains with a hypergeometric distribution test as part of the R ‘stats’ package [76].

### Generation of proteomic datasets

Lysates were prepared from ~ 50 mg of fecal material by bead beating in SDS buffer (4% SDS, 100 mM Tris-HCl, pH 8.0) using 0.15–mm diameter zirconium oxide beads. Cell debris was cleared by centrifugation (21,000×*g* for 10 min). Pre-cleared protein lysates were adjusted to 25 mM dithiothreitol and incubated at 85 °C for 10 min to further denature proteins and to reduce disulfide bonds. Cysteine residues were alkylated with 75 mM iodoacetamide, followed by a 20-min incubation at room temperature in the dark. After incubation, proteins were isolated by chloroform-methanol extraction. Protein pellets were washed with methanol, air-dried, and resolubilized in 4% sodium deoxycholate (SDC) in 100 mM ammonium bicarbonate (ABC) buffer, pH 8.0. Protein samples were quantified by BCA assay (Pierce) and transferred to a 10-kDa MWCO spin filter (Vivaspin 500; Sartorius) before centrifugation at 12,000×*g* to collect denatured and reduced proteins atop the filter membrane. The concentrated proteins were washed with 100 mM ABC (2× the initial sample volume) followed by centrifugation. Proteins were resuspended in a 1× volume of ABC before proteolytic digestion. Protein samples were digested in situ using sequencing-grade trypsin (G-Biosciences) at a 1:75 (wt/wt) ratio and incubated at 37 °C overnight. Samples were diluted with a 1× volume of 100 mM ABC, supplied with another 1:75 (wt/wt) aliquot of trypsin, and incubated at 37 °C for an additional 3 h. Tryptic peptides were then spin-filtered through the MWCO membrane and acidified to 1% formic acid to precipitate the residual SDC. The SDC precipitate was removed from the peptide solution with water-saturated ethyl acetate extraction. Samples were concentrated via SpeedVac (Thermo Fisher), and peptides were quantified by BCA assay (Pierce) before LC-MS/MS analysis.

Twelve micrograms of each peptide sample was analyzed by automated 2D LC-MS/MS using a Vanquish UHPLC with autosampler plumbed directly in-line with a Q Exactive Plus mass spectrometer (Thermo Scientific). A 100-μm inner diameter (ID) triphasic back column [RP-SCX-RP; reversed-phase (5 μm Kinetex C18) and strong-cation exchange (5 μm Luna SCX) chromatographic resins; Phenomenex] was coupled to an in-house pulled, 75 μm ID nanospray emitter packed with 30 cm Kinetex C18 resin. Peptides were autoloaded, desalted, separated, and analyzed across four successive salt cuts of ammonium acetate (35, 50, 100, and 500 mM), each followed by a 105-min organic gradient. Mass spectra were acquired in a data-dependent mode with the following parameters: a mass range of 400 to 1500 m/z; MS and MS/MS resolution of 35 K and 17.5 K, respectively; isolation window = 2.2 m/z with a 0.5-m/z isolation offset; unassigned charges and charge states of + 1, + 5, + 6, + 7, and + 8 were excluded; dynamic exclusion was enabled with a mass exclusion window of 10 ppm and an exclusion duration of 45 s.

MS/MS spectra were searched against custom-built databases composed of the concatenated sequenced metagenome-derived predicted proteomes from all time-points, the human reference proteome from UniProt, common protein contaminants, and reversed-decoy sequences using Proteome Discover 2.2 (Thermo Scientific), employing the CharmeRT workflow [77]. Peptide spectrum matches (PSMs) were required to be fully tryptic with two miscleavages, a static modification of 57.0214 Da on cysteine (carbamidomethylated) residues, and a dynamic modification of 15.9949 Da on methionine (oxidized) residues. False-discovery rates (FDRs), as assessed by matches to decoy sequences, were initially controlled at 1% at the peptide level. To alleviate the ambiguity associated with shared peptides, proteins were clustered into protein groups by 100% identity for microbial proteins and 90% amino acid sequence identity for human proteins using USEARCH [78]. FDR-controlled peptides were then quantified according to the chromatographic area under the curve (AUC) and mapped to their respective proteins. Peptide intensities were summed to estimate protein-level abundance based on peptides that uniquely mapped to one protein group. Protein abundance distributions were then normalized across samples using InfernoRDN [79], and missing values were imputed to simulate the mass spectrometer’s limit of detection using Perseus [80] as annotated in the Reactome database [81].

## Supplementary Information

**Additional file 1.**

**Additional file 2.**

**Additional file 3.**

**Additional file 4.**

**Additional file 5.**

**Additional file 6.**

**Additional file 7.**

**Additional file 8.**

**Additional file 9.**

**Additional file 10.**

**Additional file 11.**

**Additional file 12.**

**Additional file 13.**

**Additional file 14.**

## Data Availability

The datasets supporting the conclusions of this article are available in the NCBI BioProject repository, PRJNA471744 https://www.ncbi.nlm.nih.gov/bioproject/?term=PRJNA471744, the Short Read Archive (SRA) SRR5420274 to SRR5420297, https://www.ncbi.nlm.nih.gov/sra/?term=SRR5420274, and https://github.com/patrickwest/c_parapsilosis_analysis. Candida genomes and sequencing reads from infant 06 are available in the NCBI BioProject repository PRJNA717139. Further, Candida genomes are also available via https://ggkbase.berkeley.edu/project_groups/candida_genomes (sign in as a user is required).
