# Supplementary material for: Genetic and behavioral adaptation of Candida parapsilosis to the microbiome of hospitalized infants revealed by in situ genomics, transcriptomics, and proteomics
Source: Microbiome. 2021 Jun 21;9:142. doi: 10.1186/s40168-021-01085-y (PMC8215838; doi:10.1186/s40168-021-01085-y)
Supplement: Supplementary file 2 — Additional file 1. [file 40168_2021_1085_MOESM2_ESM.pdf]

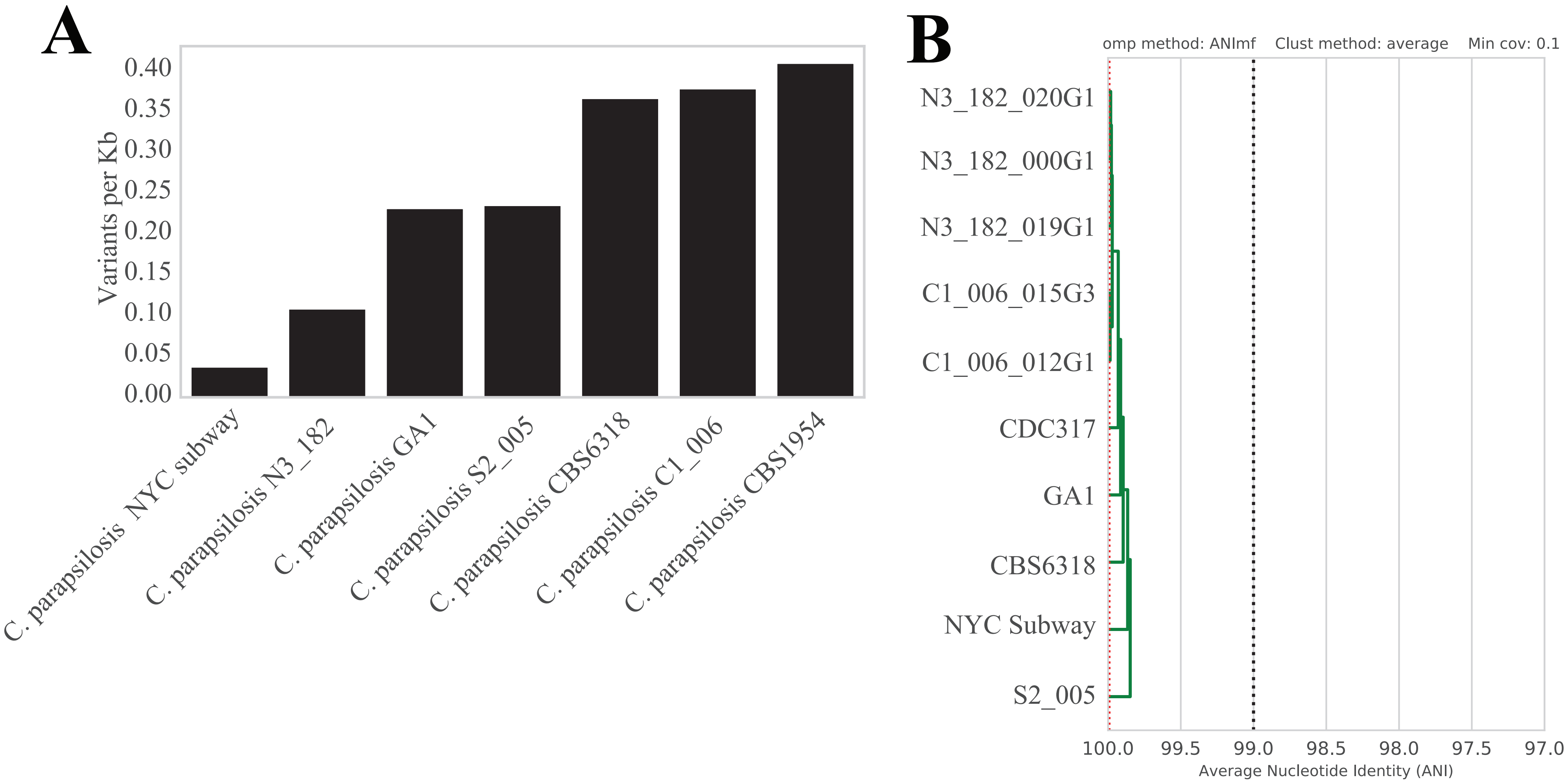

Figure S1: **Candida parapsilosis heterozygosity and ANI comparisons display low C. parapsilosis heterozygosity, even in a metagenome context, and a possible hospital associated population.** (A) C. parapsilosis heterozygosity for various strains as measured by heterozygous variants per kb.(B) ANI comparisons for each C. parapsilosis strain. Comparisons made using dRep with the ‘compare_wf’ setting. If multiple genomes were assembled for the same strain from different time points, all assembled genomes were included in this analysis.
