# Supplementary material for: Genetic and behavioral adaptation of Candida parapsilosis to the microbiome of hospitalized infants revealed by in situ genomics, transcriptomics, and proteomics
Source: Microbiome. 2021 Jun 21;9:142. doi: 10.1186/s40168-021-01085-y (PMC8215838; doi:10.1186/s40168-021-01085-y)
Supplement: Supplementary file 3 — Additional file 2. [file 40168_2021_1085_MOESM3_ESM.pdf]

**A**

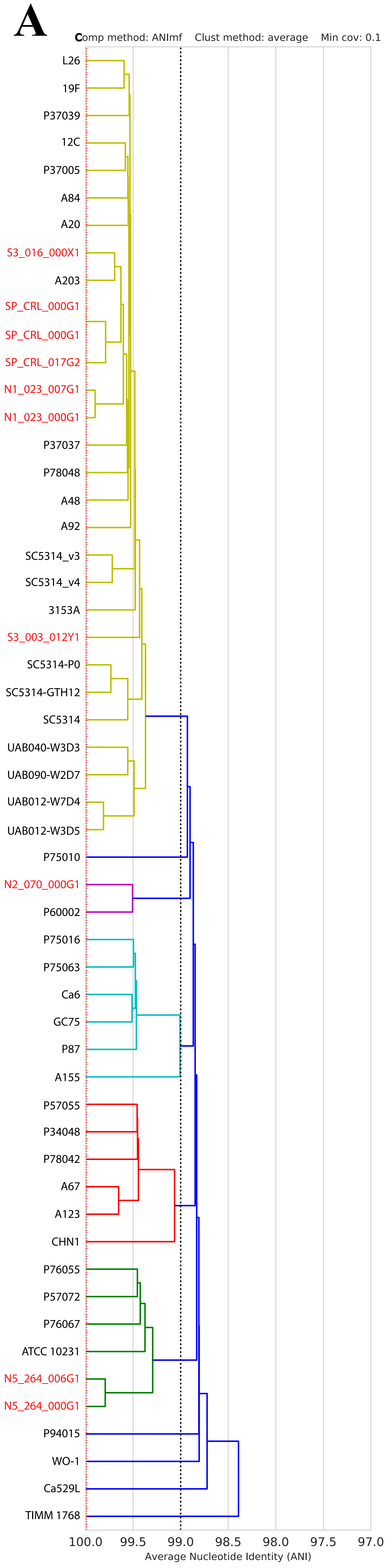


**B**

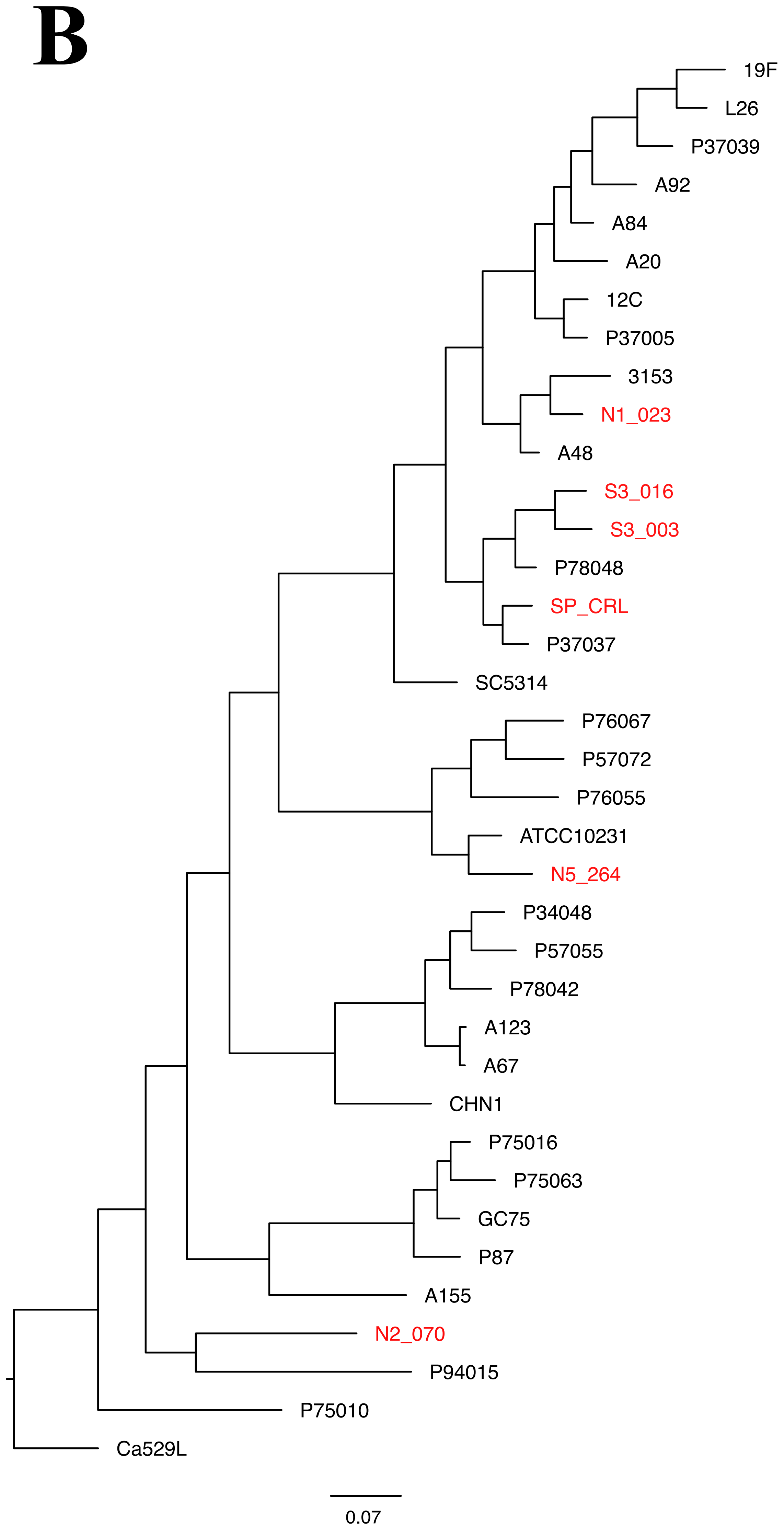


**Figure S2: Phylogenetic and ANI comparisons of C. albicans genomes show no clear hospital associated population.** (A) ANI comparisons for each C. albicans strain. All publically available C. albicans genomes from NCBI were included. Genomes assembled in this study are highlighted in red. If multiple genomes were assembled for the same strain from different time points, all assembled genomes were included in this analysis. (B) A phylogenetic tree of C. albicans strains constructed from concatenated SNVs. Strains with genomes assembled in this study are highlighted in red. All publically available C. albicans genome read sets on NCBI are included in this analysis.
