# Supplementary material for: Genetic and behavioral adaptation of Candida parapsilosis to the microbiome of hospitalized infants revealed by in situ genomics, transcriptomics, and proteomics
Source: Microbiome. 2021 Jun 21;9:142. doi: 10.1186/s40168-021-01085-y (PMC8215838; doi:10.1186/s40168-021-01085-y)
Supplement: Supplementary file 4 — Additional file 3. [file 40168_2021_1085_MOESM4_ESM.pdf]

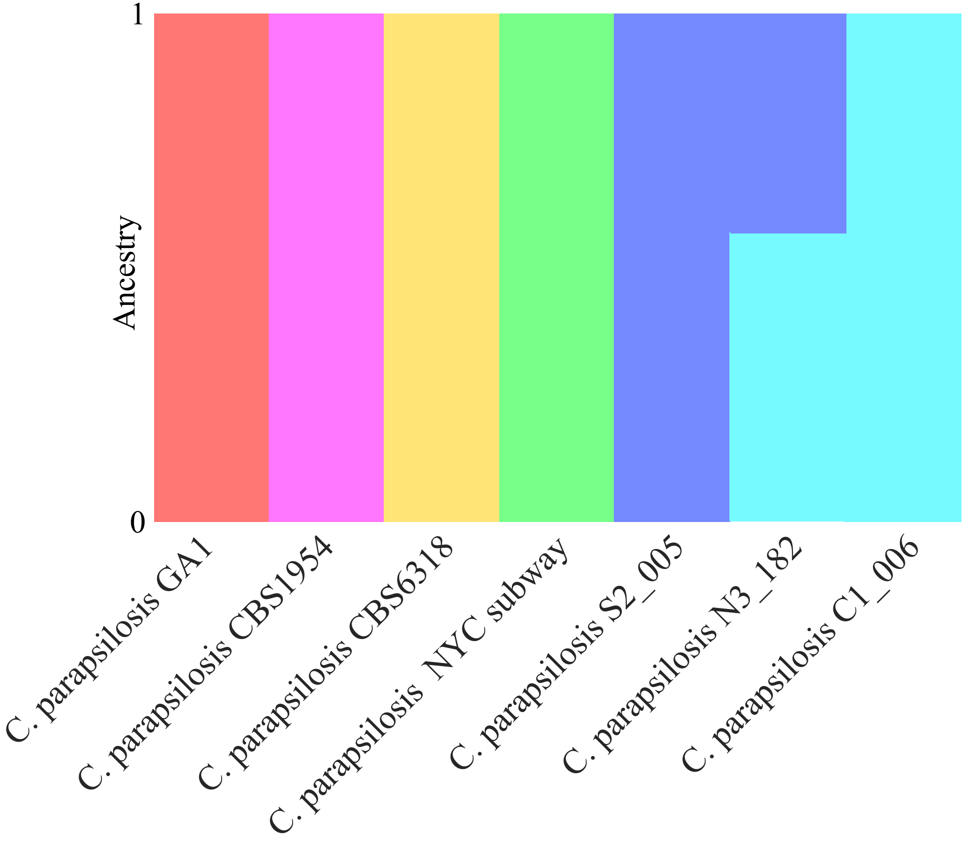

Figure S3: Population structure analysis of C. parapsilosis strain genomes performed with ADMIXTURE (Alexander et al. 2011) reveals possible admixture in strain N3_182. Each color represents a different ancestral population (N=6) and the proportion of a color in each strain represents how much of the variation in its genome is attributed to that particular ancestral population. All strains except N3_182 are predicted to have a separate ancestral population.
