# Supplementary material for: Genetic and behavioral adaptation of Candida parapsilosis to the microbiome of hospitalized infants revealed by in situ genomics, transcriptomics, and proteomics
Source: Microbiome. 2021 Jun 21;9:142. doi: 10.1186/s40168-021-01085-y (PMC8215838; doi:10.1186/s40168-021-01085-y)
Supplement: Supplementary file 6 — Additional file 5. [file 40168_2021_1085_MOESM6_ESM.pdf]

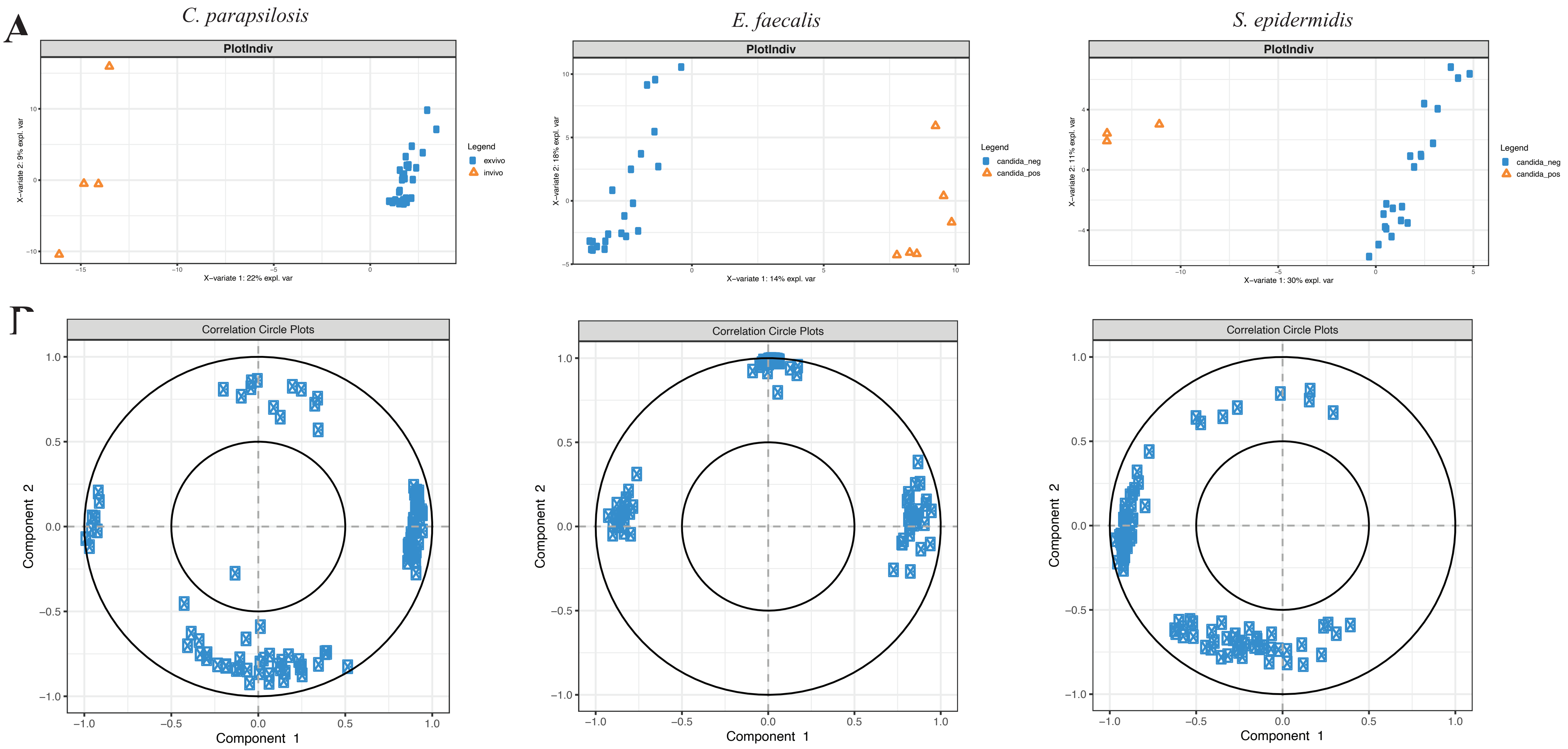

Figure S5: sPLS-DA important feature selection. (A) Separation of sample categories (in situ vs culture and Candida+ vs Candida-) based on the selected number features and visualized using the first two components of the sPLS-DA. (B) Visualization of the correlation of selected features. Features projected in the same direction are correlated, with greater distance from the origin depicting a stronger correlation.
