# Supplementary material for: Genetic and behavioral adaptation of Candida parapsilosis to the microbiome of hospitalized infants revealed by in situ genomics, transcriptomics, and proteomics
Source: Microbiome. 2021 Jun 21;9:142. doi: 10.1186/s40168-021-01085-y (PMC8215838; doi:10.1186/s40168-021-01085-y)
Supplement: Supplementary file 7 — Additional file 6. [file 40168_2021_1085_MOESM7_ESM.pdf]

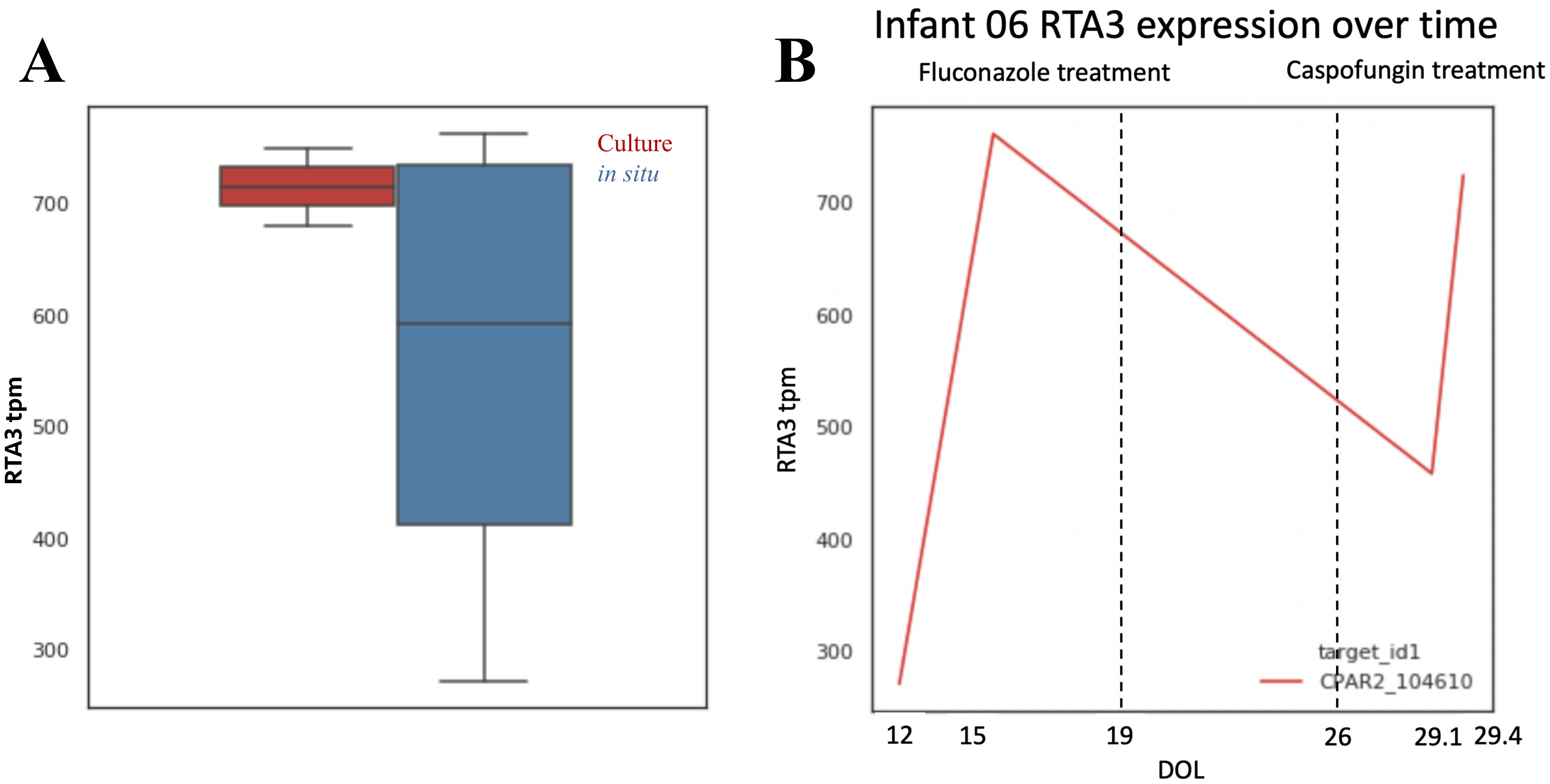

Figure S6: Expression of RTA3 in strain C1_006 shows no significant difference in expression between culture and in situ settings nor over time in infant 06 following fluconazole treatment. (A) Boxplots of RTA3 expression values for strain C1_006 both in a culture setting and in situ. (B) Time course measurement of RTA3 expression for strain C1_006 measured within infant 06 fecal samples. Fluconazole treatment was administered to infant 06 at DOL 19 and caspofungin treatment at DOL 26.
