# Supplementary material for: Genetic and behavioral adaptation of Candida parapsilosis to the microbiome of hospitalized infants revealed by in situ genomics, transcriptomics, and proteomics
Source: Microbiome. 2021 Jun 21;9:142. doi: 10.1186/s40168-021-01085-y (PMC8215838; doi:10.1186/s40168-021-01085-y)
Supplement: Supplementary file 8 — Additional file 7. [file 40168_2021_1085_MOESM8_ESM.pdf]

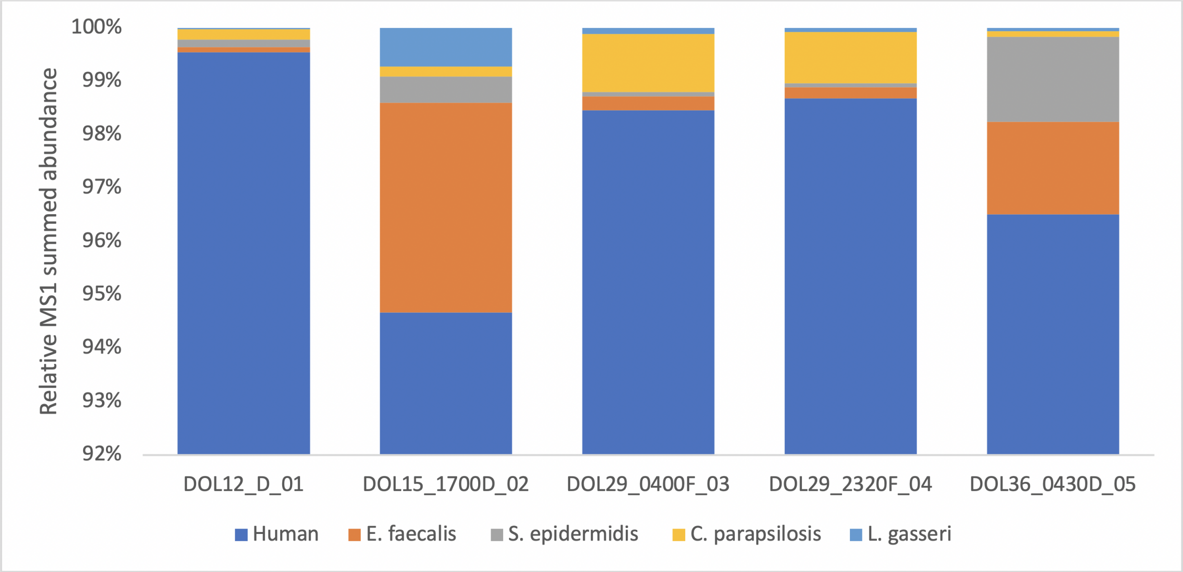

Figure S7: Organismal relative abundance based on summed protein abundances. In addition to highly abundant host proteins, protein evidence of C. parapsilosis and bacterial members was detected in all sampling time points. All members were established by day of life 12 and persisted across time.
