# Supplementary figures and images for: Genetic and behavioral adaptation of Candida parapsilosis to the microbiome of hospitalized infants revealed by in situ genomics, transcriptomics, and proteomics
Source: Microbiome. 2021 Jun 21;9:142. doi: 10.1186/s40168-021-01085-y (PMC8215838; doi:10.1186/s40168-021-01085-y)

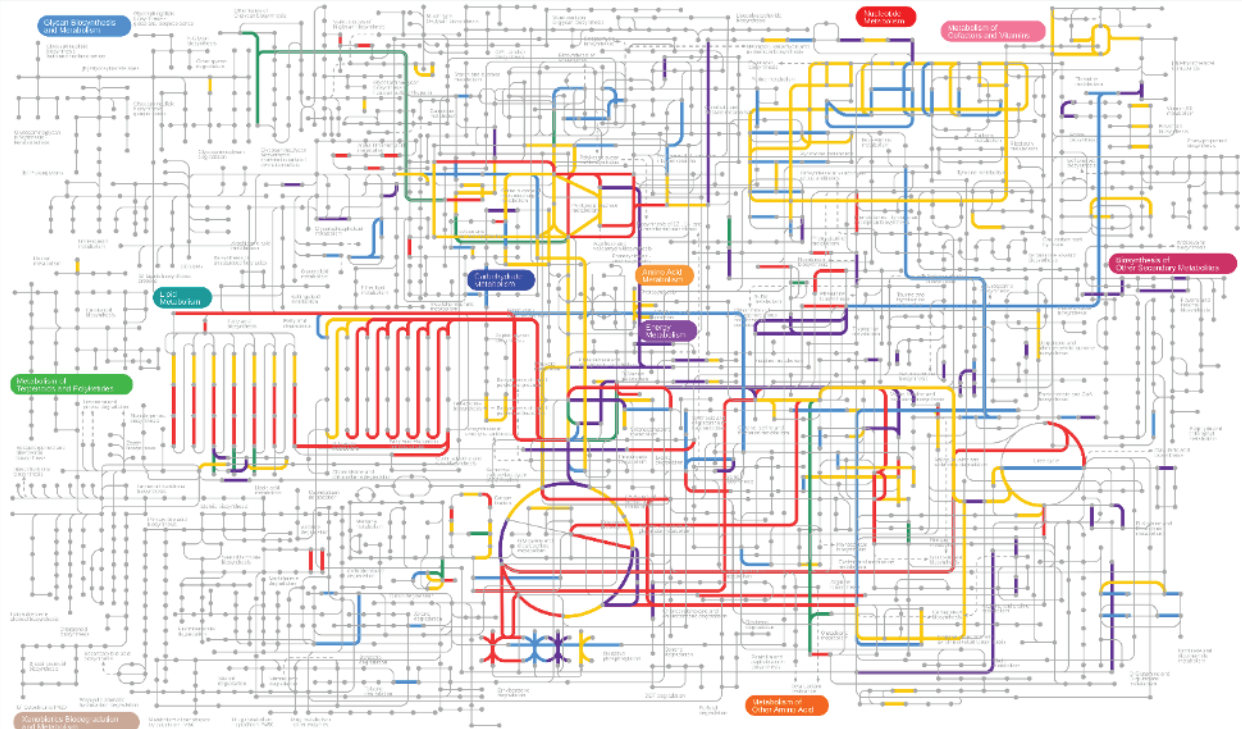

Supplement: Supplementary file 9 — Additional file 8. [file 40168_2021_1085_MOESM9_ESM.pdf]
