# Supplementary material for: Genetic and behavioral adaptation of Candida parapsilosis to the microbiome of hospitalized infants revealed by in situ genomics, transcriptomics, and proteomics
Source: Microbiome. 2021 Jun 21;9:142. doi: 10.1186/s40168-021-01085-y (PMC8215838; doi:10.1186/s40168-021-01085-y)
Supplement: Supplementary file 10 — Additional file 9. [file 40168_2021_1085_MOESM10_ESM.pdf]

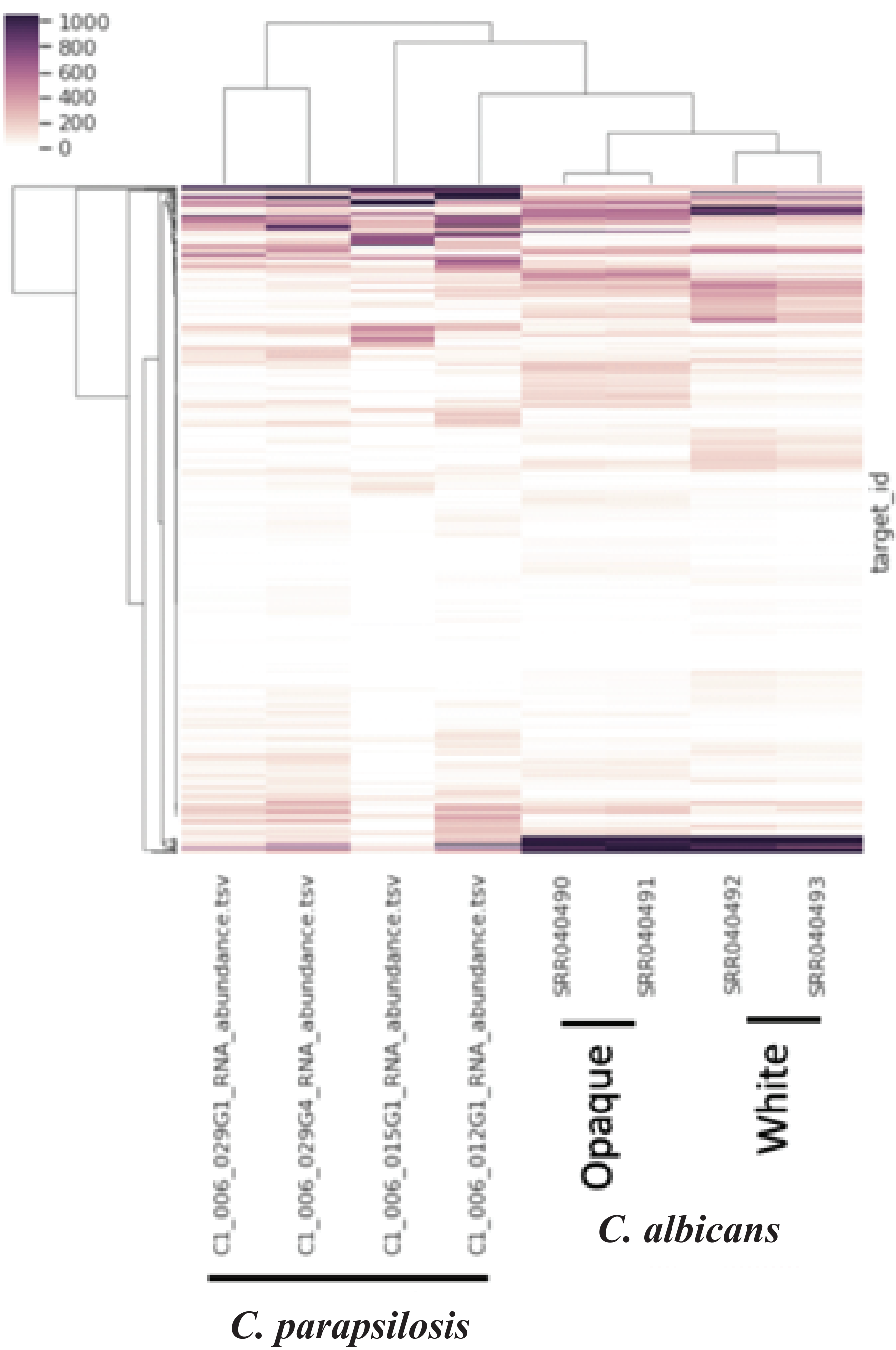


**Figure S9: C. parapsilosis in situ transcriptomes show more variance than that observed between C. albicans white and opaque phenotypes.** Samples are hierarchically clustered, with top bars reflecting how similar samples are to one another. Y axis represents only transcripts differentially expressed between white and opaque phenotypes identified in Tuch et al. 2010. C. parapsilosis orthologs for each C. albicans transcript were identified with orthofinder.
